# Ionospheric Anomalies Related to the (M = 7.3), August 27, 2012, Puerto Earthquake, (M = 6.8), August 30, 2012 Jan Mayen Island Earthquake, and (M = 7.6), August 31, 2012, Philippines Earthquake: Two-Dimensional Principal Component Analysis

**DOI:** 10.1155/2013/271513

**Published:** 2013-06-05

**Authors:** Jyh-Woei Lin

**Affiliations:** Department of Earth Science, National Cheng Kung University, No. 1 University Road, Tainan 701, Taiwan

## Abstract

Two-dimensional principal component analysis (2DPCA) and principal component analysis (PCA) are used to examine the ionospheric total electron content (TEC) data during the time period from 00:00 on August 21 to 12: 45 on August 31 (UT), which are 10 days before the M = 7.6 Philippines earthquake at 12:47:34 on August 31, 2012 (UT) with the depth at 34.9 km. From the results by using 2DPCA, a TEC precursor of Philippines earthquake is found during the time period from 4:25 to 4:40 on August 28, 2012 (UT) with the duration time of at least 15 minutes. Another earthquake-related TEC anomaly is detectable for the time period from 04:35 to 04:40 on August 27, 2012 (UT) with the duration time of at least 5 minutes during the Puerto earthquake at 04: 37:20 on August 27, 2012 (UT) (*M*_*w*_ = 7.3) with the depth at 20.3 km. The precursor of the Puerto earthquake is not detectable. TEC anomaly is not to be found related to the Jan Mayen Island earthquake (*M*_*w*_ = 6.8) at 13:43:24 on August 30, 2012 (UT). These earthquake-related TEC anomalies are detectable by using 2DPCA rather than PCA. They are localized nearby the epicenters of the Philippines and Puerto earthquakes.

## 1. Introduction

Principal component analysis (PCA) has been used to detect the ionospheric total electron content (TEC) precursors regardless of nonearthquake TEC disturbances from Lin's statistical work [7] about PCA. From his work, PCA assigns large principal eigenvalue, that is, principal eigenvalue >0.5 in a normalized set to the earthquake-related TEC anomaly (TEC precursor). When a matrix with the high dimension is transformed into the PCA domain, this matrix will be simultaneously reduced to the low dimension with minimum loss of data information in the transformed process. Therefore computing time is saved, and principal eigenvalue can represent main characteristics of data [1].

In this paper, both two-dimensional principal component analysis (2DPCA) and principal component analysis (PCA) are performed to detect TEC anomaly related to three large earthquakes. The first earthquake is; the Puerto earthquake (*M*_*w*_ = 7.3) occurred at 04:37:20 on August 27, 2012 (UT) with the epicenter of (12.278°N, 88.528°W) and the depth at 20.3 km. The second earthquake is the Jan Mayen Island earthquake (*M*_*w*_ = 6.8) occurred at 13:43:24 on August 30, 2012 (UT) with the epicenter of (71.461°N, 10.919°W) and the depth at 9.9 km. The third earthquake is; the Philippines earthquake (*M*_*w*_ = 7.6) occurred at 12:47:34 on August 31, 2012 (UT) with the epicenter of (10.838°N, 126.704°E) and the depth at 34.9 km (US Geological Survey). The examined ionospheric total electron content (TEC) data are during the time period from 00:00 on August 21 to 12:45 on August 31, 2012 (UT), which are 10 days before the Philippines earthquake. The TEC precursors were usually found in 5 days before the large earthquakes [2], and therefore the previous examined period is selected for having the TEC data of 6 days before the Puerto earthquake, and the TEC precursor of this earthquake is possible to be detected. The TEC data are acquired from the NASA Global Differential GPS (GDGPS) system.

## 2. Method

### 2.1. PCA and 2DPCA

2DPCA performing is essentially PCA on the rows of the data if each row is viewed as a computational unit. For 2DPCA, let data be represented by a matrix *B* with the dimension of *m* × *n*. Linear projection of the matrix *B* is considered as follows [3–5]:

(1) $$\begin{array}{c} y=Bx. \end{array}$$

Here *x* is an *n* dimensional project axis, and *y* is the projected feature of this data on *x* called principal component vector. *E* is mean:

(2) $$\begin{array}{c} W_{x}=E\left(y-Ey\right){\left(y-Ey\right)}^{T}. \end{array}$$

Here *W*_*x*_  is the covariance matrix of the project feature vector.

The trace of *W*_*x*_  is defined,

(3) $$\begin{array}{c} J\left(x\right)=\mathrm{tr}\left(W_{x}\right) \end{array}$$

(4) tr⁡(Wx)=tr⁡{xTSx}, where  S=E[(B−EB)T(B−EB)].

The matrix *W*_*x*_ is called covariance matrix. The alternation criterion is expressed by *J*(*x*) = tr⁡(*x* ^*T*^ *Wx*), where the data inner-scatter matrix *Wx* is computed in a straightforward manner by

(5) Wx=1m∑k=1m(Bk−B−)T(Bk−B−),        where  B−=1m∑1mBk.

The vector *x* maximizing (4) corresponds to the largest (principal) eigenvalue of *W*_*x*_ which represented the main characteristics of data. 2DPCA is another version of PCA. Therefore large principal eigenvalue of 2DPCA also indicates earthquake-related TEC anomaly. If the PCA is used to transform a matrix with low dimension into the PCA domain, then the dimension of this matrix in the PCA domain will be too small after reducing and become small sample size (SSS) data. Therefore the SSS problem will be caused by using PCA. The SSS problem causes larger data reconstruction error when data in the PCA domain are transformed back to their original domain, and corresponding principal eigenvalue is not very precise to represent main characteristics of data. The SSS problem will be removed when performing 2DPCA due to a different algorithm from the PCA. More detailed contents about the algorithm of 2DPCA can be read from the studies of Fukunnaga [3], Kong et al. [4], and Sanguansat [5].

### 2.2. TEC Data Processing Using PCA and 2DPCA

**Figure 1:**
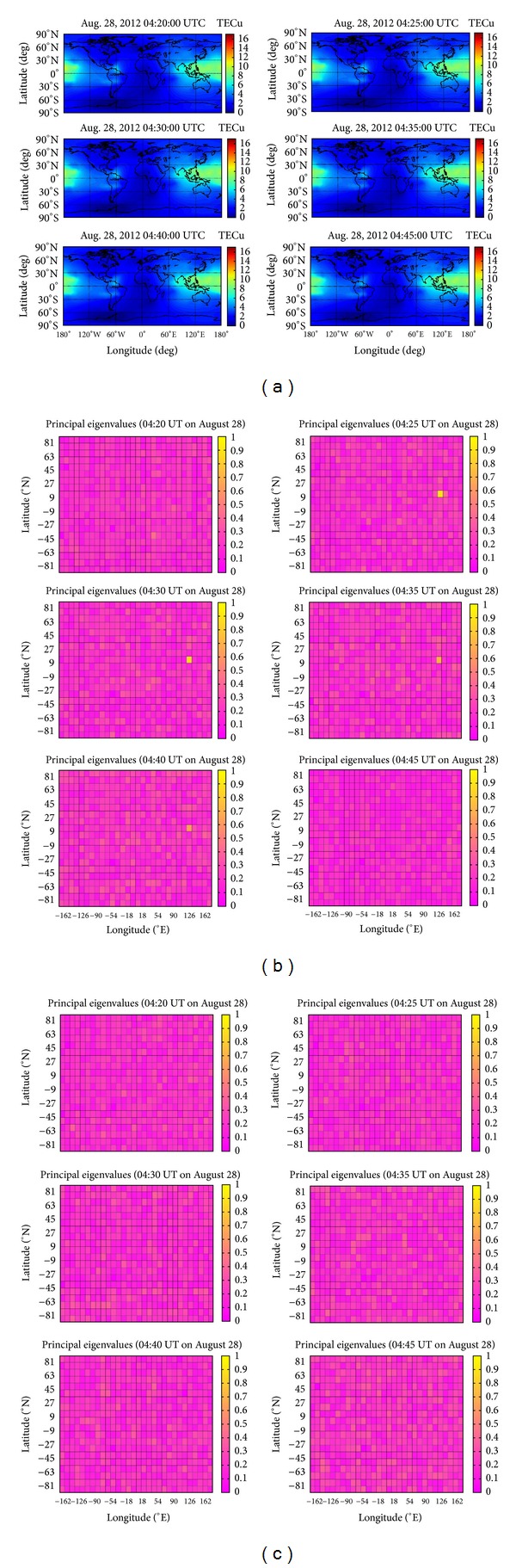
(a) The figures show the GIMs during the time period from 04:20 to 04:45 on 28 August 2012 (UT). (b) The figures give a color-coded scale of the magnitudes of principal eigenvalues of 2DPCA corresponding to Figure 1(a). The color within a grid denotes the magnitude of a principal eigenvalue corresponding to Figure 1(a), so that there are 600 principal eigenvalues assigned for 600 grids in each small map, respectively. (c) The figures give a color-coded scale of the magnitudes of principal eigenvalues of PCA corresponding to Figure 1(a).

The previouly examined TEC data are processed by using PCA and 2DPCA, and no earthquake-related anomaly is found. Only during the time period from 04:20 to 04:45 on August 28, 2012 (UT) related to the Philippines earthquake and the time period from 04:35 to 04:45 on August 27, 2012 (UT) related to the Puerto earthquake, earthquake-related TEC anomalies are detectable by using 2DPCA. Therefore the procedure of TEC data processing during the previous time periods is represented in this study.  Figure 1(a) shows the Global ionospheric TEC maps (GIMs) during the time period from 04:20 to 04:45 UT on August 28, 2012. The TEC data of each GIM in Figure 1(a) are divided into 600 smaller grids 12° in longitude and 9° in latitude, respectively. The resolution of the TEC data for this GPS system is 5 and 2.5 degrees in latitude and longitude, respectively [6], and therefore the 8 TEC data are used to compute in each grid. These 8 TEC data form the matrix with the dimensions 2 × 4 in (1) and the matrix of Lin's work [7] in order to perform 2DPA and PCA, respectively.  Figure 1(b) gives a color-coded scale of the magnitudes of principal eigenvalues of 2DPCA corresponding to Figure 1(a). From the figures, 600 principal eigenvalues are assigned. A TEC anomaly with a large principal eigenvalue is given nearby the epicenter of the Philippines earthquake during the time period from 04:25 to 04:40 UT on August 28, 2012 (UT). It is a TEC precursor for the Philippines earthquake on August 31, 2012. For comparison,Figure 1(c) gives a color-coded scale of the magnitudes of principal eigenvalues of PCA corresponding to Figure 1(a). TEC anomaly related to earthquake with a large principal eigenvalue is not found.

**Figure 2:**
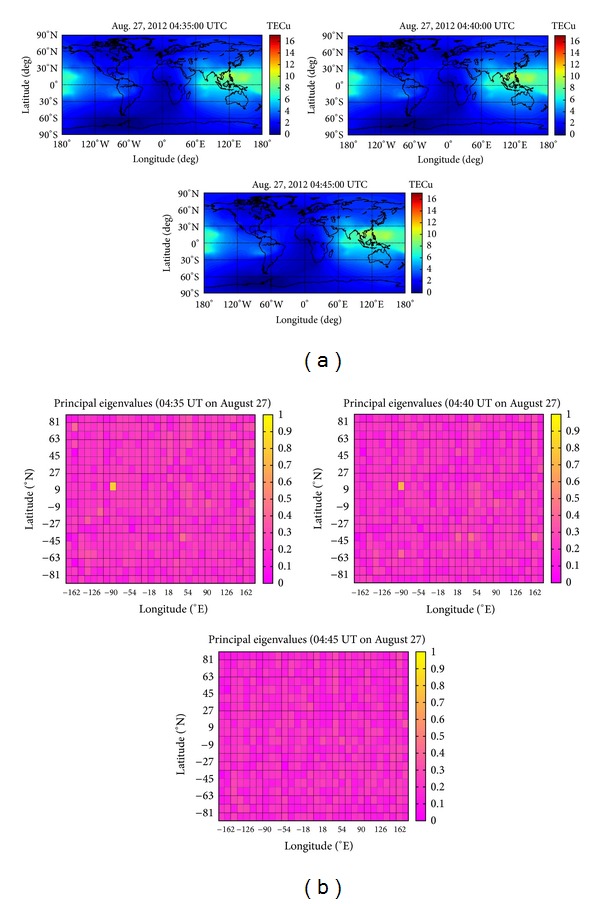
(a) The figures show the GIMs during the time period from 04:35 to 04:45 on 27August 2012 (UT). (b) The figures give a color-coded scale of the magnitudes of principal eigenvalues of 2DPCA corresponding to Figure 2(a). (c) The figures give a color-coded scale of the magnitudes of principal eigenvalues of PCA corresponding to Figure 2(a).

**Figure 3:**
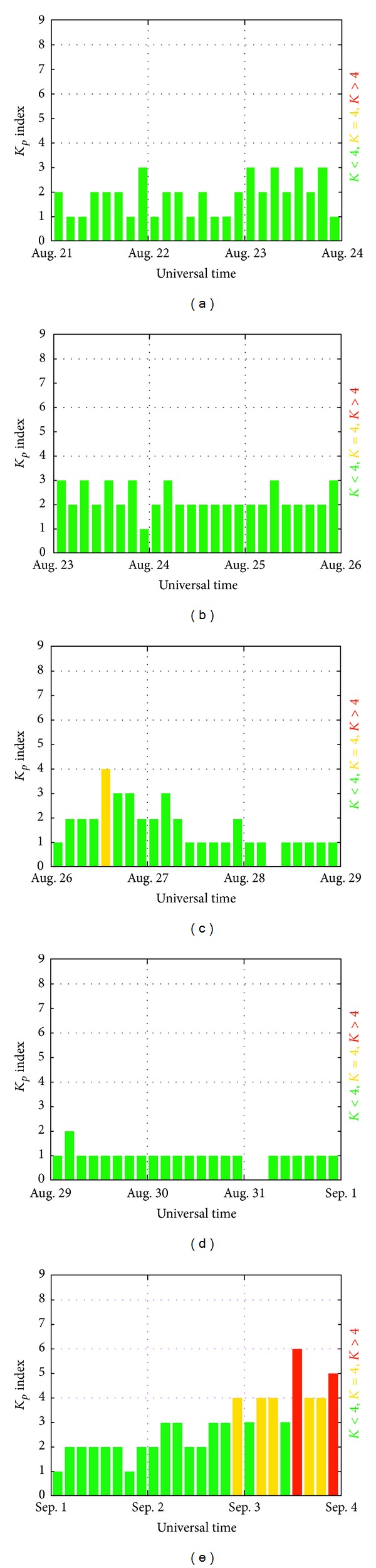
The figures show the *K*_*p*_ indices from 21 August to 03 Septembers 2012 (UT) (NOAA Space Weather Prediction Center).

TEC data of the GIMs in Figure 2(a) during the time period from 04:35 to 04:45 on August 27, 2012 (UT) are processed with previous same analysis method by using 2DPCA and PCA. The results of 2DPCA and PCA are shown in Figure 2(b) and Figure 2(c), respectively. The TEC anomaly related to the Puerto earthquake is found nearby the epicenter during the time period 04:35 to 04:40 on August 27, 2012 (UT) by using 2DPCA. TEC anomaly related to other small earthquakes during the examined time period is not detectable. From previous results, TEC anomaly related to earthquake is not found by using PCA. The reason should be the inputted matrix in previous TEC data processing is the SSS data due to the low dimension.  Figure 3 shows the *K*_*p*_ indices (August 21 to September 03, 2012 UT) on August 27 and 28, 2012 (UT) are small indicating that geomagnetic activity can not be responsible for the TEC anomaly.

## 3. TEC Data Testing during Other Time Periods

**Figure 4:**
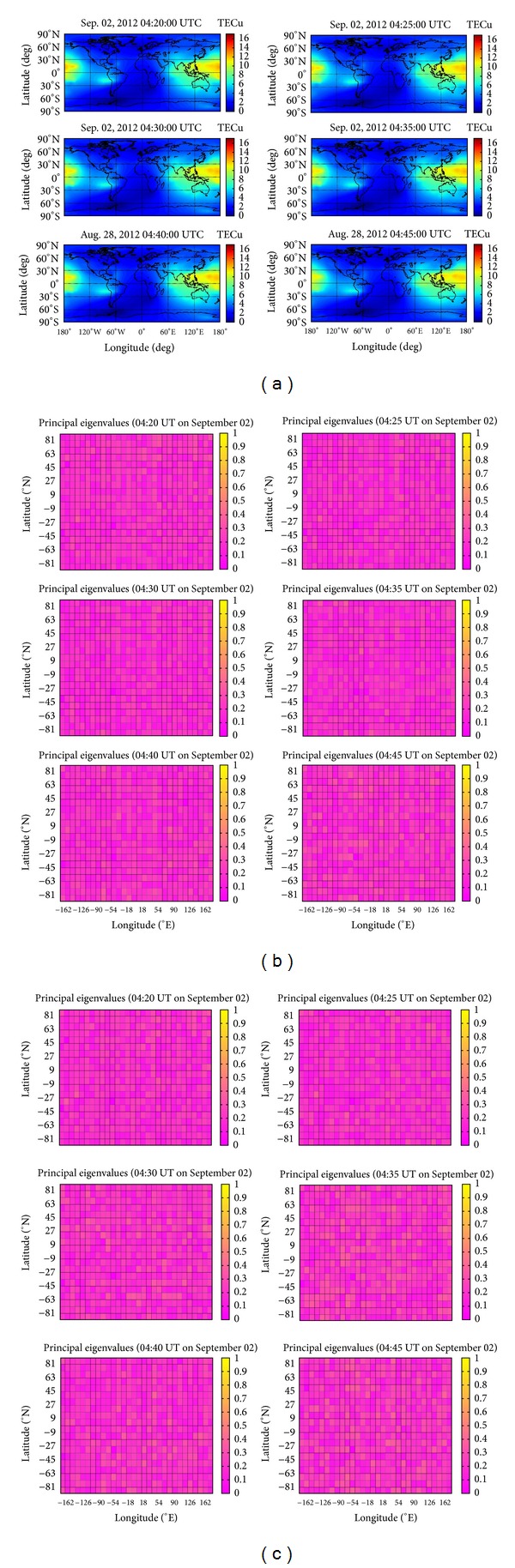
(a) The figures show the GIMs at the time 04:20 to 04:45 on 02 September 2012 (UT). (b) The figures give a color-coded scale of the magnitudes of principal eigenvalues of 2DPCA corresponding to Figure 4(a). (c) The figures give a color-coded scale of the magnitudes of principal eigenvalues of PCA corresponding to Figure 4(a).

For comparison, the TEC data of the GIMs during the time period from 04:20 to 04:45 on September 02, 2012 (UT) after the three earthquakes in Figure 4(a) are examined using 2DPCA and PCA to see if TEC anomalies are detectable due to geomagnetic quiet days (Figure 3). The results of 2DPCA and PCA are shown in Figures 4(b) and 4(c) with the same TEC data processing described in Section 2.2, respectively. No earthquake-associated TEC anomaly is evident with large principal eigenvalue. The results have confirmed the TEC anomalies on August 27 and 28, 2012 (UT) to be associated with the earthquakes.

## 4. Discussion

2DPCA is able to detect a TEC precursor nearby the epicenter of Philippines earthquake during the time period from 04:25 to 04:40 on August 28, 2012 (UT) with the duration time of at least 15 minutes. The TEC anomaly related to the Puerto earthquake nearby the epicenter is found during the time period 04:35 to 04:40 on August 27, 2012 (UT) with the duration time of at least 5 minutes. 2DPCA has the ability to detect the clear TEC anomalies related to the large earthquakes in this study rather than PCA. The reason which caused the TEC anomaly related to the Puerto earthquake should be shock acoustic wave. The results of Afraimovich et al.'s work [8] have shown that the shock acoustic waves due to occurring of the earthquakes have affected ionosphere for two earthquakes in Turkey on August 17, and November 12, 1999 and in Southern Sumatra on June 04, 2000, and they have found the ionospheric anomaly related to these earthquakes due to shock acoustic wave to be 180–390 s. Therefore compared with the Afraimovich et al.'s results on the TEC anomaly related to the Puerto earthquake with the duration time of at least 5 minutes (300 s), 2DPCA has shown its credibility to estimate the duration time of the earthquake-associated TEC anomaly and then confirmed that the reason of this TEC anomaly should be shock acoustic wave due to the earthquake. TEC anomaly related to the Jan Mayen Island earthquake on August 30, 2012 is not detectable. An argument always exists; principal eigenvalue could not indicate true ionospheric variations or situation. However, if a mathematical index can indicate an ionospheric precursor, then the aim of earthquake precursor research is already to be satisfied. More detailed corresponding precursor research can be examined from the reports of VAN group (Varotsos, Caesar Alexopoulos, and Kostas Nomikos) and some studies [9–14].

## 5. Conclusion

A TEC precursor has been detectable for the Philippines earthquake during the time period from 04:25 to 04:40 on August 28 (UT), and its duration time was at least 15 minutes. An earthquake-related TEC anomaly during the Puerto earthquake was found during time period from 04:35 to 04:40 on August 27, 2012 (UT), and its duration time was at least 5 minutes. TEC anomaly related to Jan Mayen Island earthquake was not detectable.
